# Supplementary material for: Calcium-vesicles perform active diffusion in the sea urchin embryo during larval biomineralization
Source: PLoS Comput Biol. 2021 Feb 22;17(2):e1008780. doi: 10.1371/journal.pcbi.1008780 (PMC7932551; doi:10.1371/journal.pcbi.1008780)
Supplement: S1 Fig — These images show average vesicle speed and size for each vesicle tracked, in both skeletogenic cells (A) and ectoderm (B). A trendline fit is shown for each region, in orange for control and in blue for VEGFR inhibition. In both regions and in all experimental condition Pearson’s R2 < 0.013, showing no correlation between vesicle size and velocity. (PDF) [file pcbi.1008780.s001.pdf]

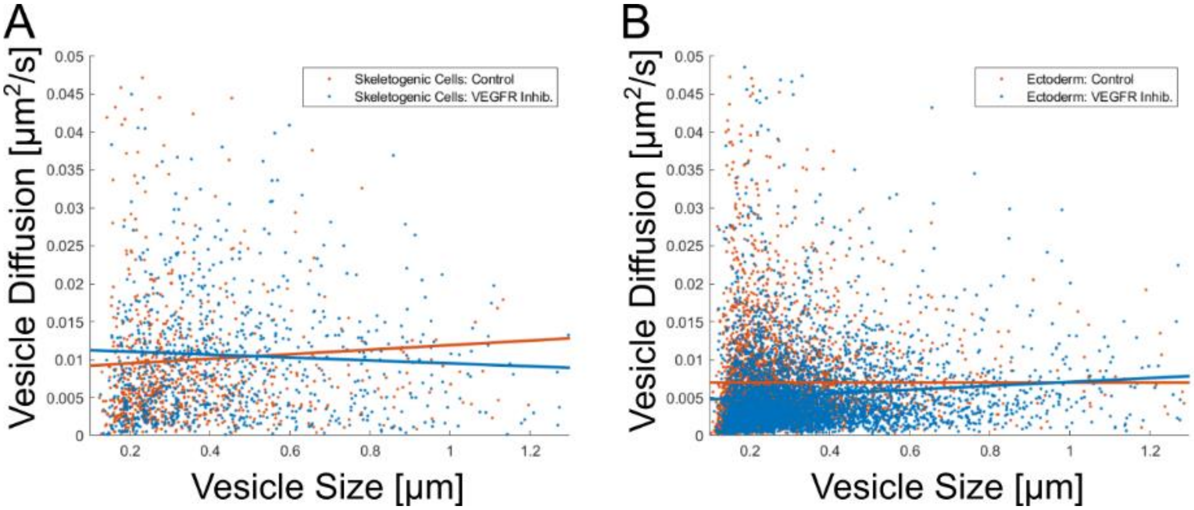

Supplementary Figure 1 Vesicle instantaneous speed vs. size scatter plot comparison. These images show average vesicle speed and size for each vesicle tracked, in both skeletogenic mesodermal (A) and ectodermal (B) regions. A trendline fit is shown for each region, in orange for control and in blue for VEGFT inhibition. In both regions and in all experimental condition Pearson's $R^2 < 0.013$, showing no correlation between vesicle size and velocity.
