# Supplementary material for: Calcium-vesicles perform active diffusion in the sea urchin embryo during larval biomineralization
Source: PLoS Comput Biol. 2021 Feb 22;17(2):e1008780. doi: 10.1371/journal.pcbi.1008780 (PMC7932551; doi:10.1371/journal.pcbi.1008780)
Supplement: S2 Fig — Shown are histograms of vesicle instantaneous speed for every track in control (skeletogenic cell, A, ectoderm, B) and VEGFR inhibition (skeletogenic cell, C, ectoderm, D). For each histogram there is a single peak, making it unlikely that there are multiple different motion types (e.g. progressive movement vs. vesicle pausing observed in motor-guided movement). (PDF) [file pcbi.1008780.s002.pdf]

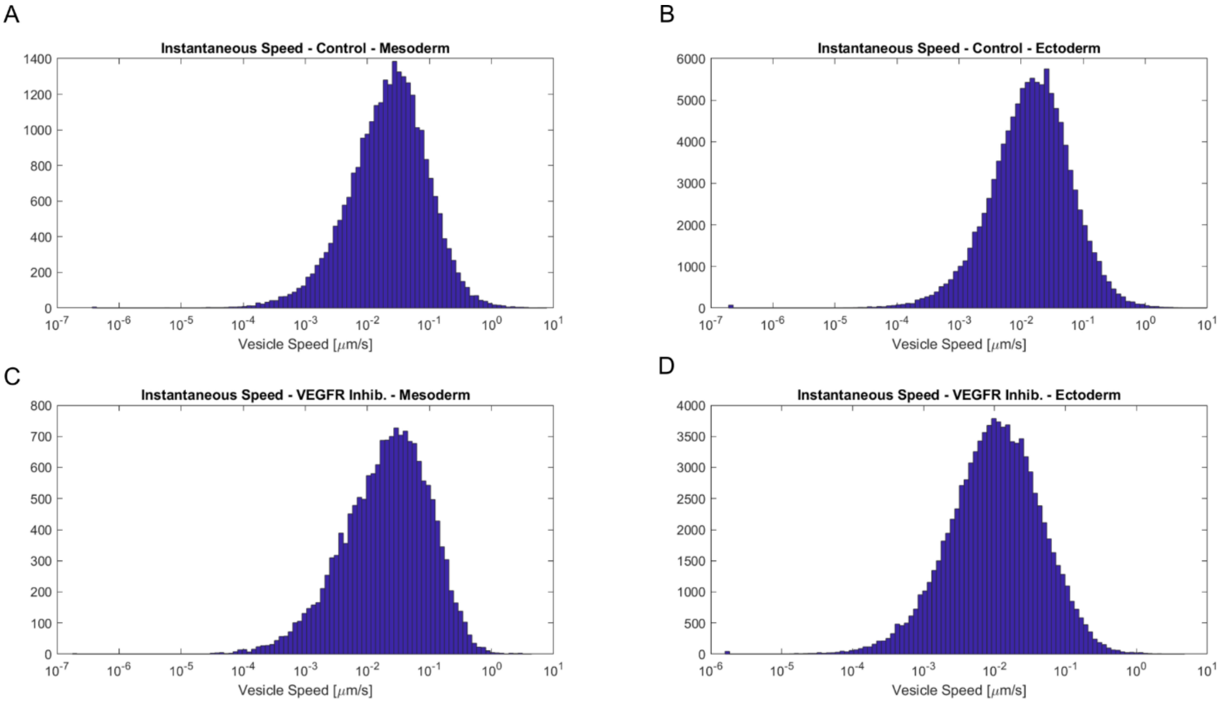

Supplementary Figure 2 Vesicle instantaneous speed histograms. Histograms of vesicle instantaneous speed for every track in control (A, B) and VEGFR inhibition (C, D) are shown. For each histogram there is a single peak, making it unlikely that there are multiple different motion types (*e.g.* progressive movement vs. vesicle pausing observed in motor-guided movement).
