# Supplementary material for: Calcium-vesicles perform active diffusion in the sea urchin embryo during larval biomineralization
Source: PLoS Comput Biol. 2021 Feb 22;17(2):e1008780. doi: 10.1371/journal.pcbi.1008780 (PMC7932551; doi:10.1371/journal.pcbi.1008780)
Supplement: S3 Fig — The plot is an example of this computing the diffusion coefficient as the slope of the linear relationship between the mean-square displacement and time for a single vesicle track. (PDF) [file pcbi.1008780.s003.pdf]

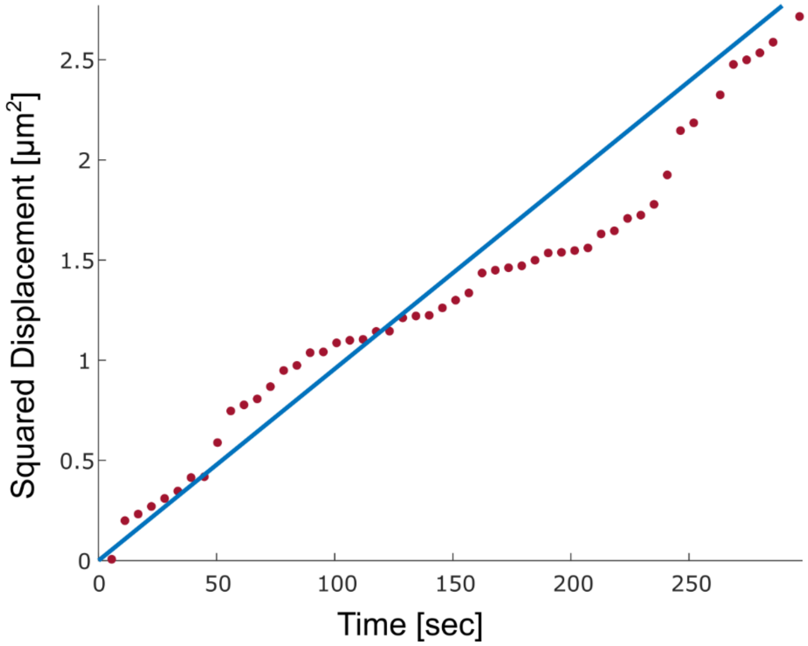

Supplementary Figure 3 The diffusion coefficient is calculated as the linear fit of the ratio between squared displacement and time. The plot is an example of this computing the diffusion coefficient as the slope of the linear relationship between squared displacement and time for a single vesicle track.
