# Supplementary material for: Calcium-vesicles perform active diffusion in the sea urchin embryo during larval biomineralization
Source: PLoS Comput Biol. 2021 Feb 22;17(2):e1008780. doi: 10.1371/journal.pcbi.1008780 (PMC7932551; doi:10.1371/journal.pcbi.1008780)
Supplement: S4 Fig — These images show vesicle diffusion rate relative to average vesicle size for each vesicle tracked, in both skeletogenic mesodermal (A) and ectodermal (B) regions. A linear regression fit is shown for each region, orange for control and blue for VEGFR inhibition. In both regions the Pearson’s R2 < 0.007, indicating that there is practically no correlation between vesicle size and diffusion coefficient. (PDF) [file pcbi.1008780.s004.pdf]

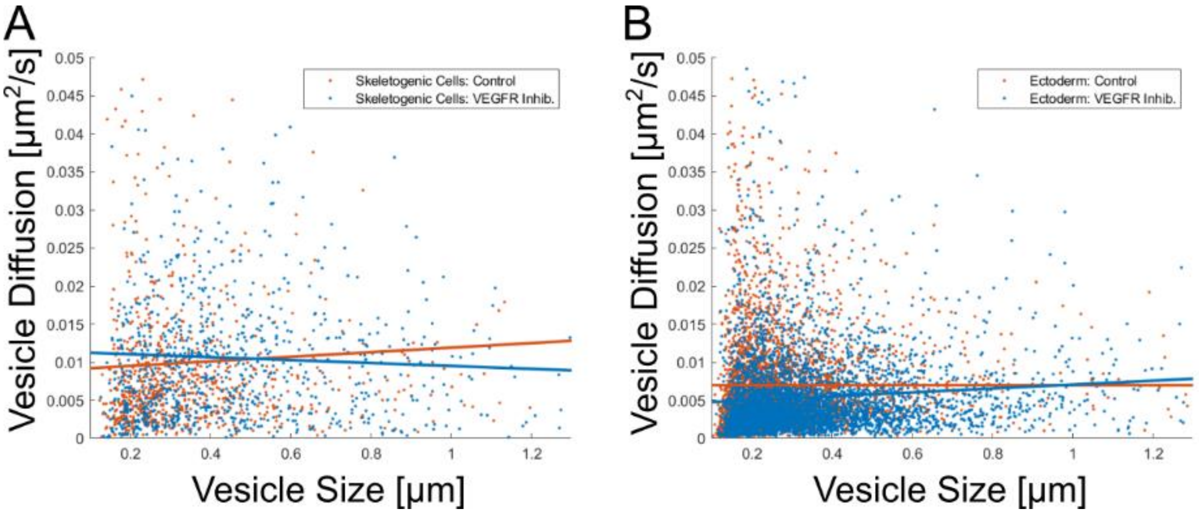

Supplementary Figure 4 Vesicle diffusion coefficient vs. size scatter plot comparison. These images show vesicle diffusion rate relative to average vesicle size for each vesicle tracked, in both skeletogenic mesodermal (A) and ectodermal (B) regions. A linear regression fit is shown for each region, orange for control and blue for VEGFR inhibition. In both regions the Pearson's $R^2 < 0.007$, indicating that there is practically no correlation between vesicle size and diffusion rate.
